# Effects of protein concentration and beta-adrenergic agonists on ruminal bacterial communities in finishing beef heifers

**DOI:** 10.1371/journal.pone.0296407

**Published:** 2024-02-29

**Authors:** Alison P. Pfau, Madison T. Henniger, Kendall L. Samuelson, Kristin E. Hales, Clint A. Löest, Mike E. Hubbert, Amanda K. Lindholm-Perry, Amanda M. Egert-McLean, Katie M. Mason, Elizabeth A. Shepherd, Brynn H. Voy, Phillip R. Myer

**Affiliations:** 1 Department of Animal Science, University of Tennessee, Knoxville, Tennessee, United States of America; 2 Department of Agricultural Sciences, West Texas A&M University, Canyon, Texas, United States of America; 3 Department of Animal and Food Sciences, Texas Tech University, Lubbock, Texas, United States of America; 4 Department of Animal and Range Sciences, New Mexico State University, Las Cruces, New Mexico, United States of America; 5 Department of Animal and Range Sciences, Clayton Livestock Research Center, New Mexico State University, Clayton, New Mexico, United States of America; 6 USDA, ARS, U.S. Meat Animal Research Center, Clay Center, Nebraska, United States of America; University of Illinois, UNITED STATES

## Abstract

To improve animal performance and modify growth by increasing lean tissue accretion, beef cattle production has relied on use of growth promoting technologies such as beta-adrenergic agonists. These synthetic catecholamines, combined with the variable inclusion of rumen degradable (RDP) and undegradable protein (RUP), improve feed efficiency and rate of gain in finishing beef cattle. However, research regarding the impact of beta-adrenergic agonists, protein level, and source on the ruminal microbiome is limited. The objective of this study was to determine the effect of different protein concentrations and beta-adrenergic agonist (ractopamine hydrochloride; RAC) on ruminal bacterial communities in finishing beef heifers. Heifers (*n* = 140) were ranked according to body weight and assigned to pens in a generalized complete block design with a 3 × 2 factorial arrangement of treatments of 6 different treatment combinations, containing 3 protein treatments (Control: 13.9% CP, 8.9% RDP, and 5.0% RUP; High RDP: 20.9% CP, 14.4% RDP, 6.5% RUP; or High RUP: 20.9% CP, 9.7% RDP, 11.2% RUP) and 2 RAC treatments (0 and 400 mg/day). Rumen samples were collected via orogastric tubing 7 days before harvest. DNA from rumen samples were sequenced to identify bacteria based on the V1-V3 hypervariable regions of the 16S rRNA gene. Reads from treatments were analyzed using the packages ‘phyloseq’ and ‘dada2’ within the R environment. Beta diversity was analyzed based on Bray-Curtis distances and was significantly different among protein and RAC treatments (P < 0.05). Alpha diversity metrics, such as Chao1 and Shannon diversity indices, were not significantly different (P > 0.05). Bacterial differences among treatments after analyses using PROC MIXED in SAS 9 were identified for the main effects of protein concentration (P < 0.05), rather than their interaction. These results suggest possible effects on microbial communities with different concentrations of protein but limited impact with RAC. However, both may potentially act synergistically to improve performance in finishing beef cattle.

## 1 Introduction

To aid in addressing global food security concerns and availability to high-quality protein, the beef cattle industry must increase lean mass yield, while also increasing feed efficiency. Due to its cost-effectiveness, abundant availability, and nutritional value, byproducts from the corn milling industry have been commonly included in feed for beef cattle [1]. The ingredients and nutrient composition of finishing cattle diets have been altered by increasing the use of byproducts from the corn milling industry. The inclusion of these byproducts at high rates typically results in diets with an excess supply of dietary energy and protein [2, 3]. Research has shown limited improvements in performance when crude protein (CP) concentration in finishing diets was greater than 13% [4, 5]. However, if CP was greater than 15.5% in combination of highly degradable protein in the rumen, results demonstrated a potential metabolic cost associated with ammonia detoxification. These results indicate that protein degradability plays an important role in animal performance [6].

Developing new or improved technologies to manipulate management and nutrition of finishing cattle, with the intent of improving lean carcass yield, is the aim of continuing research. Traditionally, the use of beta-adrenergic agonists, such as ractopamine hydrochloride (RAC), has been used to improve feed efficiency, increase gain, as well as, decrease the deposition of fat in the carcass by the stimulation of adrenoreceptors localized in the muscle and adipose tissue [7–9]. RAC, marketed under the commercial trade name Optaflexx (Elanco Animal Health; Greenfield, IN), is a beta-adrenergic agonist (synthetic catecholamine) used to improve finishing cattle performance within the last 28 to 42 days prior to harvest [9]. Beta-adrenergic agonists are phenethanolamine compounds with similar characteristics to endogenous catecholamines, such as epinephrine and norepinephrine [10]. The economic benefits of beta-adrenergic agonists can be demonstrated by the quick and large uptake by the US beef industry of the supplement, with reports showing that that 85% of feedlot operators use beta-adrenergic agonists during the finishing period [2, 3].

Beta-adrenergic agonists share similarities with natural catecholamines in structural and pharmacological properties [11]. Catecholamines, such as norepinephrine, epinephrine, and dopamine, have been shown to produce an increase in Gram-negative bacterial growth [12]. The demonstration of the catecholamines ability to interact and impact bacterial growth has been studied by the examination of different mechanisms. Lyte and Bailey [12] showed that the position of the hydroxyl group on the catechol ring induced bacterial growth under specific conditions of iron restriction [13]. Iron requirements play an important role in microbial and mammalian physiology. For this reason, bacteria under poor iron environments due to the presence of lactoferrin (iron-sequestering glycoproteins) and transferrin, localized in the mammalian gut lumen, and in the plasma and throughout all internal organs respectively, will secrete siderophores and bind with the hydroxyl group of the catecholamines to obtain the iron required for growth. Freestone et al. [14] confirmed that catecholamines, such as norepinephrine, facilitate the extraction of iron from transferrin and lactoferrin for the availability of the bacteria utilization for growth [14], demonstrating effects of natural catecholamines on bacterial communities.

Animals fed diets high in rumen degradable protein (RDP) in combination with RAC improved performance compared with RAC alone [2, 3, 15, 16], highlighting the interaction between the RAC and dietary protein. However, the interaction of RAC and protein sources with microbes in the rumen is relatively unknown. It has been suggested that the supplementation with RAC could alter proteolysis processes in the rumen, affecting the potential of the rumen microbiota to utilize rumen degradable protein, and others have observed that the ratio between protein fractions of RDP and rumen undegradable protein (RUP) could increase the response to RAC [16]. Therefore, it was hypothesized that the interaction of various concentrations of degradable and undegradable protein, in combination with beta-adrenergic agonists, may affect the ruminal microbial communities in beef heifers during the finishing period. The objective of this study was to determine the effect of protein concentration and RAC supplementation on ruminal bacterial communities in finishing beef heifers.

## 2 Methods and materials

### 2.1 Animal management

This study and all procedures were approved by the New Mexico State University Institutional Animal Care and Use committee. As described in Samuelson et al. [2, 3], cattle received initial vaccines and parasiticides (Safeguard; Intervet Inc., Millsboro, DE) when they initially arrived at the Clayton Livestock Research Center, 56 days before they were weighed for the current study. Calves were weighed using a Daniels Bud Box System (AH- 10; Daniels Mfg., Ainsworth, NE). Prior to the study, calves were fed a commercial starter diet (RAMP; Cargill). After 56 days on the commercial starter diet and prior to the study initiation, a total of 140 heifers were weighed individually (423 ± 1.8 kg), ranked by body weight, vaccinated again (Vista 3; Merck Animal Health, Summit, NJ) and received a commercial growth implant (Revalor-200; Merck Animal Health). All cattle were medium framed, commercial, crossbred cattle. Based on body weight, animals were separated into 4 blocks and assigned to pens with similar average weight and standard deviation [2, 3].

### 2.2 Experimental design

The study utilized a generalized complete block design with sampling and repetitions, consisting of 48 pens and 4 blocks. There were 12 pens per block with 9 to 11 heifers per pen, and 3 heifers per pen were randomly selected for rumen fluid collection [2, 3]. Pen size was 12 × 35 m, and the feed bunk space was 11-m bunk line. Within each block, pens of heifers were randomly assigned to 1 of 6 treatments in a 2 × 3 factorial arrangement. The treatments consisted of 400 mg or 0 mg of ractopamine hydrochloride (Optaflexx, Elanco Animal Health, Greenfield, IN) per head per day supplied in steam-flaked corn-based diets with 3 different dietary protein treatments (Table 1): **CON** (13.9% CP, 8.9% RDP, and 5.0% RUP), High **RDP** (20.9% CP, 14.4% RDP, 6.5% RUP) and High **RUP** (20.9% CP, 9.7% RDP, 11.2% RUP). Prior to the treatment period, heifers were fed a standard feedlot finishing diet (CON). After sorted into pens and weighed (initial BW = 498 Kg (Block 1), 527 Kg (Block 2), 551 Kg (Block 3) and 575 ± 3.82 Kg (Block 4)), heifers were fed the dietary treatments. The animals were fed twice daily, with a treatment period of 35 days.

**Table 1:** Ingredients and nutrient composition of diets fed to finishing heifers^1^.

Ingredients	Protein Treatments^2^		
	CON	High RDP	High RUP
*Ingredient*. *% of DM*			
Corn grain, flaked	67.1	57.0	57.0
Wet corn gluten feed	18.0	18.0	14.5
Corn Stover	9.00	9.00	9.00
Soybean Meal	—	9.60	—
Corn gluten meal	—	—	14.50
Corn oil	0.90	1.40	—
Urea	1.01	1.49	—
Supplement^3^	3.99	3.51	5.00
*Nutrient Analysis*, *DM basis*			
TDN, %	84.4	84.3	84.2
CP, %	13.9	20.9	20.9
RDP, %	8.85	14.40	9.70
RUP, %	5.05	6.46	11.18
ADF, %	8.80	8.97	9.10
Ca, %	0.78	0.76	0.79
P, %	0.41	0.45	0.44
Mg, %	0.19	0.22	0.18
K, %	0.75	0.85	0.78
S, %	0.17	0.20	0.30
Na, %	0.13	0.14	0.15
Zn, mg/kg	79	80	80
Fe, mg/kg	254	251	228
Mn, mg/kg	49	51	48
Cu, mg/kg	13.70	13.50	15.70
ME, Mcal/kg ^4^	3.05	3.05	3.04
NEm, Mcal/kg^5^	2.08	2.07	2.07
NEg, Mcal/kg^6^	1.41	1.41	1.40

^1^ Information from [2, 3]

^2^ Treatments were in a 2 x 3 factorial arrangement with sampling and repetitions, with 2 levels of ractopamine hydrochloride (400 mg or 0 mg per animal daily) and 3 dietary protein treatments (CON, High RDP, or High RUP).

^3^ Contain dried distillers’ grains with soluble, limestone, salt, trace minerals (1.8% Cu, 9.0% Zn, and 360 ppm Se; Beefmax 0510; Cargill Inc.) vitamins (1030 IU vitamin A, 500 IU vitamin D, and 5.62 IU vitamin E per kg of DM) and medicated supplement (supplied 33mg of monensin and 9.8mg of tylosin per kg of dietary DM; Elanco Animal Health, Indianapolis, IN).

^4^ ME = TDN x 4.409 x 0.82 [17].

^5^ NEm = 1.37 x ME– 0.138 x ME^2^ + 0.0105 x ME^3^–1.12 [17].

^6^ NEg = 1.42 x ME– 0.174 x ME^2^ + 0.0122 x ME^3^–1.65 [17].

The RAC (Optaflexx; Elanco Animal Health, Greenfield, IN) was administrated on top of the protein treatments in feed bunks once daily according with the doses of RAC (0 or 400 mg) per heifer assigned to 1 of 3 dietary protein treatments. The RAC was mixed with 150 g of wet corn gluten feed (SweetBran; Cargill, Inc.) for pens receiving RAC treatment, whereas pens without RAC treatment were top dressed with 150 g of wet corn gluten feed only. Specifically, RAC was administered by sprinkling/scattering the Sweetbran containing the RAC onto the diets in the feed bunk immediately after the feed truck had delivered the diet (CON, or High RDP or High RUP) to a pen of cattle. An attempt was then made to evenly disperse the RAC in the diet by using a metal rake to mix the sprinkled or “to-dressed” Sweetbran containing the RAC with the diet already in the feed bunk. Importantly, an ingredient mixing error was discovered for the High RUP treatment after cattle were transitioned to their dietary CP treatments [3]. Appropriately, data collected from three heifers were removed from this study. Further, during the treatment period, 1 animal from the CON treatment died from unknown causes, resulting in a total of 140 heifers [3].

### 2.3 Rumen sampling from the finishing heifers

Rumen fluid samples were collected from the 140 heifers by orogastric tubing 4 hours after the morning feeding on day 28 (7 days before harvest) with a metal suction strainer. Samples of rumen fluid were collected using a metal suction strainer connected via flexible tubing to a stoppered 1,000-mL side-arm Erlenmeyer flask. A vacuum was created using a squeeze bulb pump connected with flexible tubing to the side arm of the flask. To prevent saliva contamination, the first 100 mL of rumen fluid was discarded. Further, each sample was mixed in a beaker, rumen content pH was determined using a handheld pH meter, and samples were divided into 15-mL aliquots. Within each pen, rumen fluid samples were then combined, resulting in 48 total samples. Samples were stored at -80°C until further analysis.

### 2.4 DNA extraction for ruminal microbial communities

All 16S-based microbial community methods were performed as recommended by Weinroth et al. [18]. DNA was extracted from rumen fluid using the repeated bead beating plus column method described by Yu and Morrison [19]. The 15-mL rumen samples were used for distribution to new 0.2 g aliquots. Each 0.2 g sample was added to a 2-mL beaded screw cap tube, containing 0.5-mm ZR BashingBead lysis matrix for cell lysis (Zymo Research, Irvine, CA, USA). A total of 1 mL of lysis buffer (500-mM NaCl, 50-mM tris-HCl, pH 8.0, 50-mM EDTA, and 4% sodium dodecyl sulfate) was added, contents were homogenized for 3 minutes at 21 Hz, and tubes were incubated at 70°C for approximately 15 minutes before centrifugation at 16,000 x *g*. Supernatant was transferred to 2-mL tubes and 300 μL of lysis buffer was added to the original sample again for the repetition of the previous step. Following the repetition, the supernatants from individual samples were pooled.

Nucleic acid was precipitated using 260 μL of 10 M ammonium acetate added to each tube. Samples were mixed and incubated for 5 minutes on ice. Following incubation, tubes were centrifugated at 4°C for approximately 10 minutes at 16,000 x *g*. The supernatant was distributed into two tubes of 1.5 mL each, and an equal volume of isopropanol was added. Mixtures were incubated on ice for 30 minutes to precipitate DNA. Samples were centrifuged at 4°C for 15 minutes at 16,000 x *g* to pellet nucleic acid. Pellets were washed with 70% ethanol and dried for 3 minutes. The pellet was then dissolved in 100 μL of Tris-EDTA buffer and pooled for individual samples.

The QIAamp DNA Stool Mini Kit (QIAGEN, Valencia, CA, USA) was used for purification. Contamination of RNA was removed by the addition of 2 μL of DNase-free RNase (10 mg/mL) and incubated at 37°C for 15 minutes. To remove protein contamination, 15 μL of proteinase K and 200 μL of buffer AL were added. Samples were then incubated for 10 minutes at 70°C. After incubation, samples were mixed with 200 μL of 100% ethanol. The final samples were transferred to QIAamp columns (QIAGEN, Valencia, CA, USA) and centrifugated at 16,000 x g for 1 minute. Flow through was discarded and the washing process was repeated by the addition of Buffer AW1 and AW2 and centrifuged after each addition under the same conditions. The column was centrifugated at room temperature at 16,000 x g for 1 minute to completely dry the column. Following the drying process, 70 μL of Buffer AE were added to the column membrane and the samples were incubated for 2 minutes at room temperature. A volume of 30 μL of Buffer AE was added again to the column membrane and incubated again under the same conditions. To elute DNA, QIAamp column was placed into a 1.5-mL tube and centrifuged for 1 minute. DNA samples were kept at -20°C for subsequent amplification and library preparation processes.

### 2.5 DNA amplification for ruminal microbial communities

Amplicon libraries of the 16S rRNA gene for bacteria (V1-V3) were constructed as previously described [20], using primers 27F [21] and 519R [22]. Each 20 μL polymerase chain reaction (PCR) amplification reaction contained 0.5 μL Terra PCR Direct Polymerase Mix (Takara Bio, Mountain View, CA, USA) (0.625 Units), 7.5 μL nuclease-free sterile water, 10 μL 2X Terra PCR Direct Buffer, 1 μL indexed fusion primers (10 μM), and 1 μL DNA (20 to 70 ng). Thermocycling conditions were: 3 minutes of initial denaturation at 98°C, 25 cycles including 30 seconds at 98°C, 30 seconds at 55°C, and 45 seconds at 68°C. The final extension was conducted for 4 minutes at 68°C. Primers utilized for DNA amplification are listed in Table 2.

**Table 2:** DNA PCR amplification primers.

	Regions	Primers	Sequences	Source
**Bacteria**	V1—V3	27F	KRGTTYGATYNTGGCTCAG	[21]
		519R	GWRTTACCGCGGCKGCTG	[22]

Once DNA amplification was completed, the products of PCR from each sample were normalized (1 to 2 ng/ μL) using the Just-a-Plate ^™^ 96 CPR Purification and Normalization kit (Charm Biotech, MO, USA) as described by the manufacturer. After the normalization, the libraries were pooled to 10 μL per sample and purified using the Nucleospin^®^ Gel and PCR Cleanup kit (Takara Bio USA, Inc., Mountain View, CA) following the manufacture’s protocol. Libraries were analyzed for quality utilizing the BioAnalyzer 2100 (Agilent Technologies, Santa Clara, CA, USA) and DeNovix QFX Fluorometer (DeNovix dsDNA Fluorescence Quantification Assay) for quantification. Libraries were sequenced using the 2x250, v2 500-cycle kit and Illumina MiSeq sequencer (lllumina, San Diego, CA, USA).

### 2.6 DNA amplicon sequence data processing for ruminal microbial communities

Bacteria communities sequenced by the Illumina MiSeq 2x250 and resultant fastq files were processed through an R pipeline as described by Callahan and others [23]. The files were introduced into R software and open-sourced R packages ‘phyloseq’ [24] and ‘DADA2’ (Divisive Amplicon Denoising Algorithm 2) [25] for filtering, merging, and taxonomy assignment. Reads were trimmed based on quality score (Q ≥ 25) and the error expected per each read was set to two, filtering any data which did not meet these criteria [26]. DADA2 was used for accuracy in the identification of real variants, and amplicon error correction used a naïve Bayesian classifier [25, 27] Amplicon sequence variants (ASVs) were generated from DADA2, with the purpose of increasing genetic resolution in comparison to 97% operational taxonomic units (OTUs) [28, 29]. Filtered reads were merged and chimeras were removed. The SILVA v132 database [30] was used for taxonomic assignment at the genus level. Any reads mapping to *Cyanobacteria* were removed from the data.

### 2.7 Statistical analyses

Alpha-diversity and beta-diversity metrics were analyzed using the *estimate richness* and *adonis* functions from the ‘phyloseq’ and ‘vegan’ packages, respectively [31]. Alpha-diversity was calculated for observed richness (Observed), expected richness (Chao1), and richness and evenness (Shannon). Alpha diversity was calculated for bacteria and the differences among treatments were analyzed by SAS v9.4 (SAS Institute, Cary, NC, USA). The effects of diet protein, presence or absence of a beta-adrenergic agonist, and the interaction between diet protein and the beta-adrenergic agonist were analyzed using a MIXED procedure. As cattle were pulled and sampled randomly by pen over approximately 8 hours, pen was included as a random effect and significance determined at α = 0.05. Beta-diversity measures utilized a Bray-Curtis distance matrix to produce principal coordinates analyses (PCoA). Subsequently, a PERMANOVA was utilized with 999 permutations to establish significance of Bray-Curtis PCoA by the use of ‘vegan’ in R [31]. The abundance differences among the treatments for bacteria communities were calculated using PROC MIXED in SAS v9.4.

## 3 Results

### 3.1 Sequence data and diversity of rumen bacterial communities

**Fig 1:**
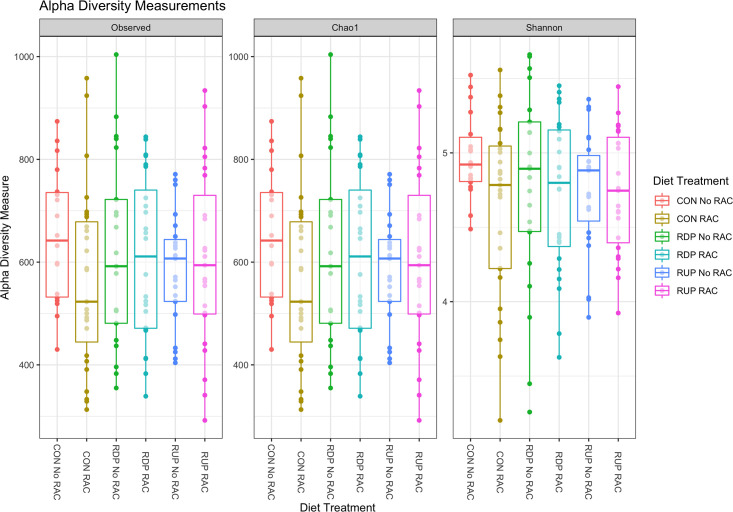
Alpha-diversity metrics for ruminal bacterial richness and evenness among the treatments described by observed, Chao1 and Shannon. Colors represent different treatment diets grouped by treatment diets of control (CON), rumen degradable protein (RDP), rumen undegradable protein (RUP), and the presence or absence of ractopamine hydrochloride (RAC).

After quality control, filtering, and processing the sequences in R software, the ruminal contents of the 48 samples contained 13,805 unique taxa. Alpha- and beta- diversity measures indicated the richness, evenness, and diversity among the bacterial communities. The alpha-diversity indices for each treatment were not significantly different (*P* > 0.05; Fig 1).

**Fig 2:**
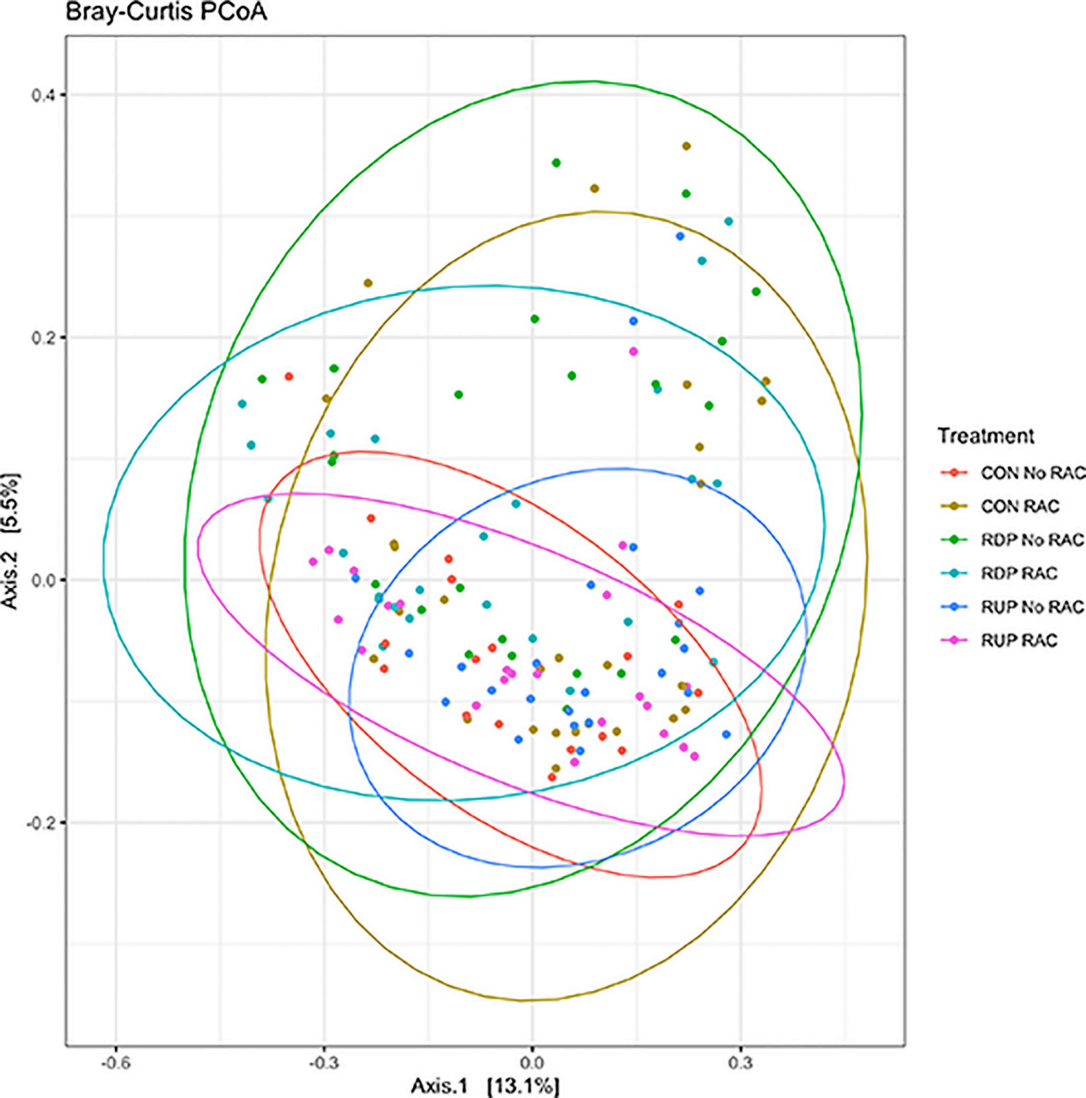
Beta-diversity measurement for bacterial communities using a Bray-Curtis PCoA grouped by treatment diets of control (CON), rumen degradable protein (RDP), rumen undegradable protein (RUP), and the presence or absence of ractopamine hydrochloride (RAC). Ellipses and points with same color represent samples within treatments groups (*P* < 0.05). Clusters indicate similarities among treatments groups.

Data was normalized and analyzed by PCoA to determine separation based on diversity among samples. Visually, there was no indication of sample clustering and indicated some degree of treatment overlap (Fig 2). A PERMANOVA was utilized to assess differences in beta diversity, demonstrating differences among the 6 treatments (*P* < 0.05). Following, dispersion estimates calculated for the Bray-Curtis distance matrix were significant (*P* < 0.05), indicating that the samples were influenced by differences in dispersion among samples, and not true differences.

### 3.2 Bacterial community composition

**Fig 3:**
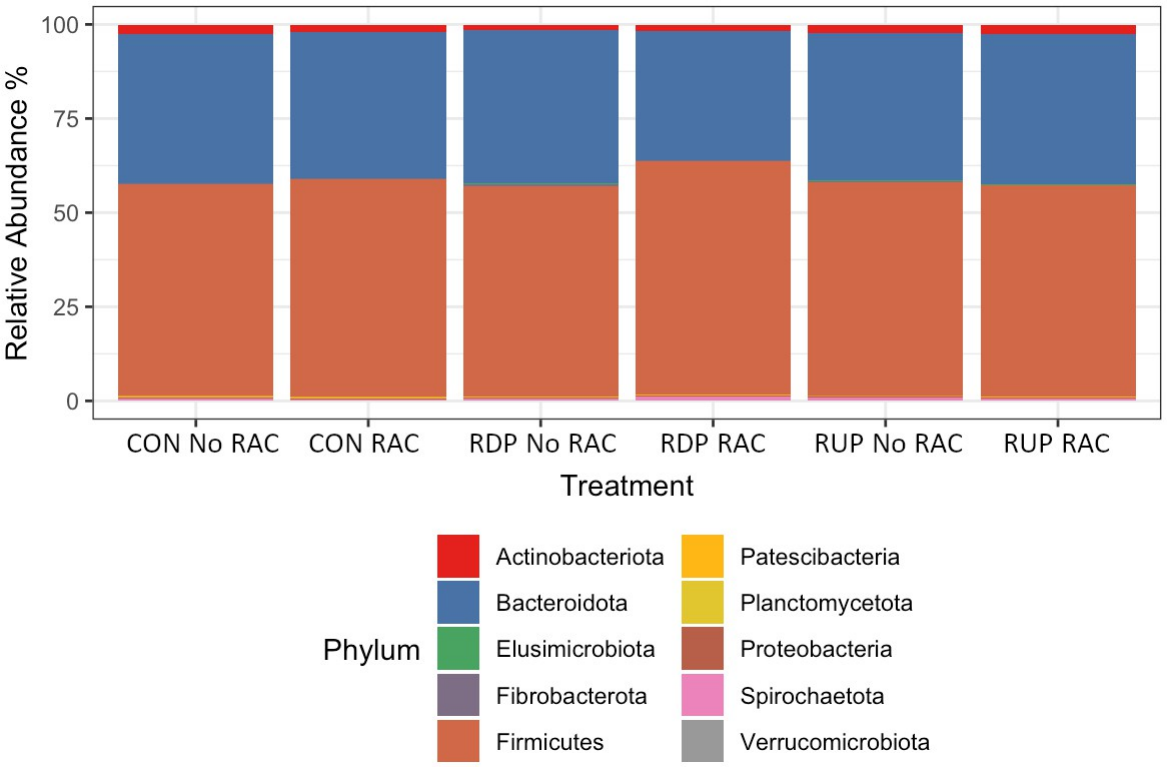
Overall phyla relative abundance identified in the ruminal bacterial communities, grouped by the treatment diets of control (CON), rumen degradable protein (RDP), rumen undegradable protein (RUP), and the presence or absence of ractopamine hydrochloride (RAC).

**Fig 4:**
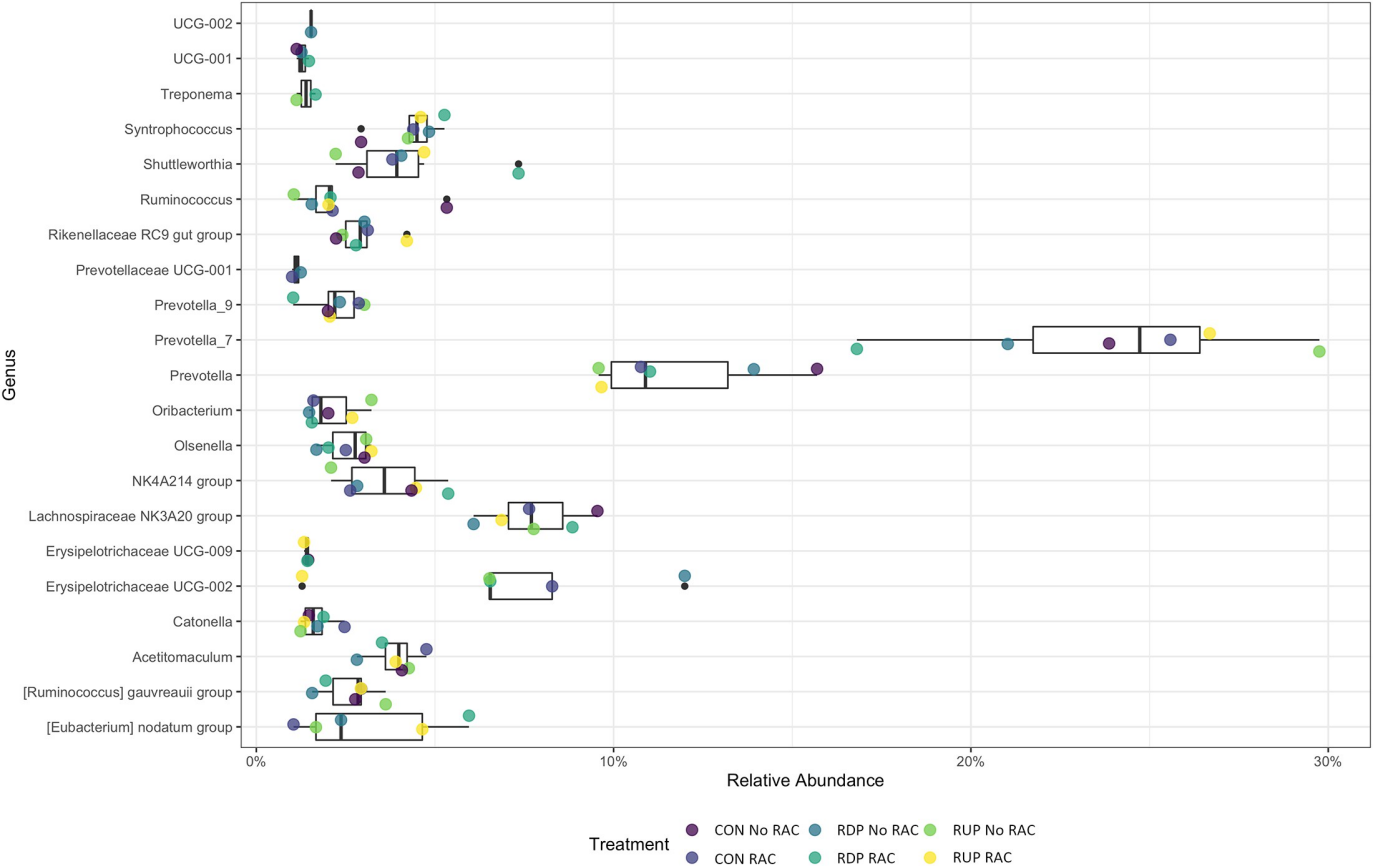
Taxonomic profile of the 99% genera relative abundance of the ruminal bacterial communities among the treatments. The boxplot represents the mean across all samples and different colored points represent different treatments. Purple represents the control diet (CON), blue represents the rumen degradable protein diet (RDP), and yellow represents the rumen undegradable protein diet (RUP).

The abundances of the top 10 phyla present in rumen bacterial samples revealed the most predominant bacterial phyla identified in the samples. Within all treatments, the top phyla of Bacteroidetes and Firmicutes were 30–35% and 57–60%, respectively (Fig 3). The remaining phyla in the rumen bacterial samples represented less than 1% of the remaining reads. Patescibacteria and Actinobacteria accounted for the less dominant phyla (greater than 0.5% but less than 1%). At the genus level, the most abundant genera were primarily *Prevotella_7* and *Prevotella* (16.4% and 8.1%, respectively) followed by Lachnospiraceae *NK3A20 group* and Erysipelotrichaceae *UCG-002* (8.5% and 7.6%, respectively; Fig 4).

**Fig 5:**
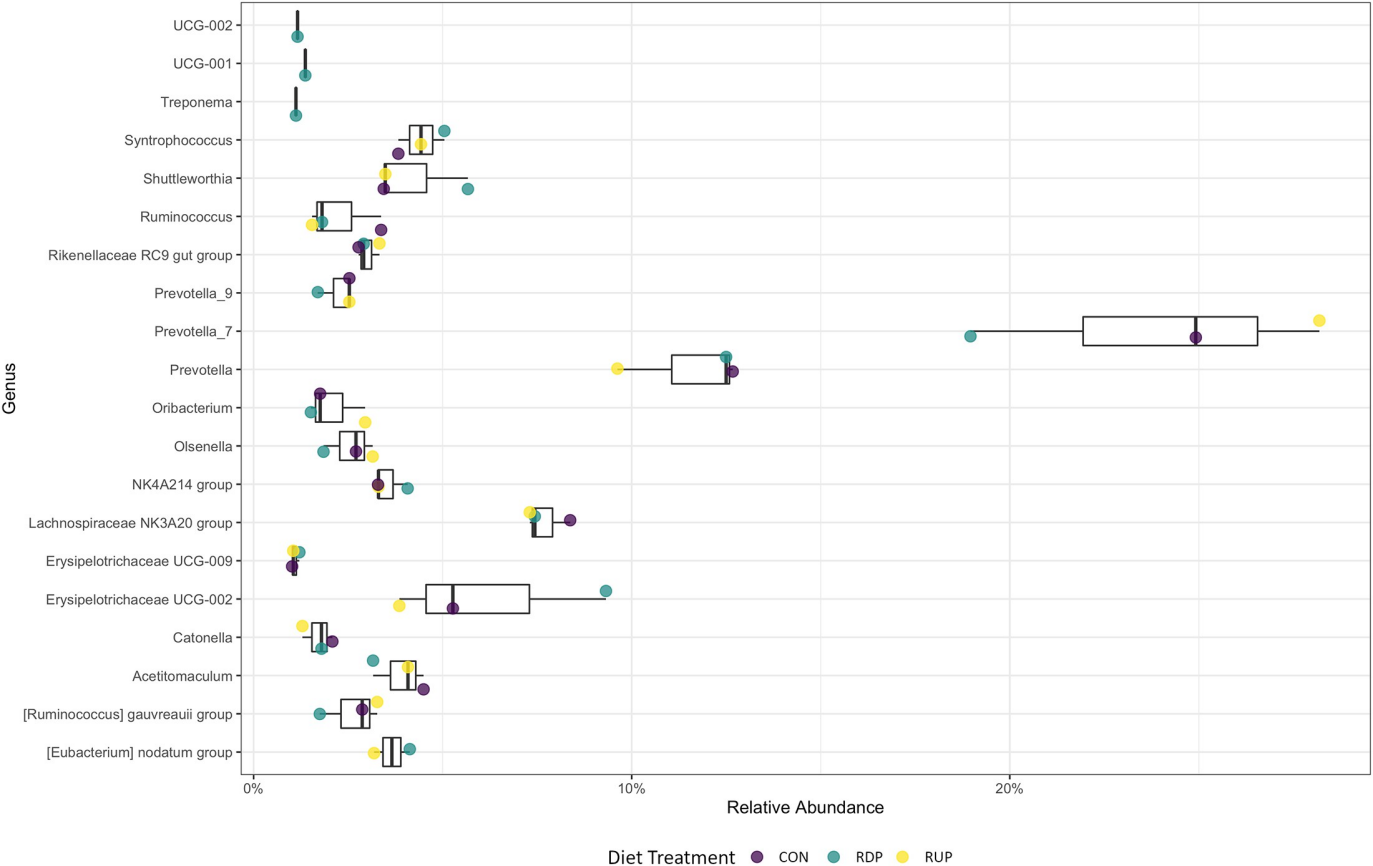
Taxonomic profile of the 99% genera relative abundance of the ruminal bacterial communities between the protein concentration. The boxplot represents the genus-level abundances across all samples and different colored points represent different diets. Purple represents the control diet (CON), blue represents the rumen degradable protein diet (RDP), and yellow represents the rumen undegradable protein diet (RUP).

**Fig 6:**
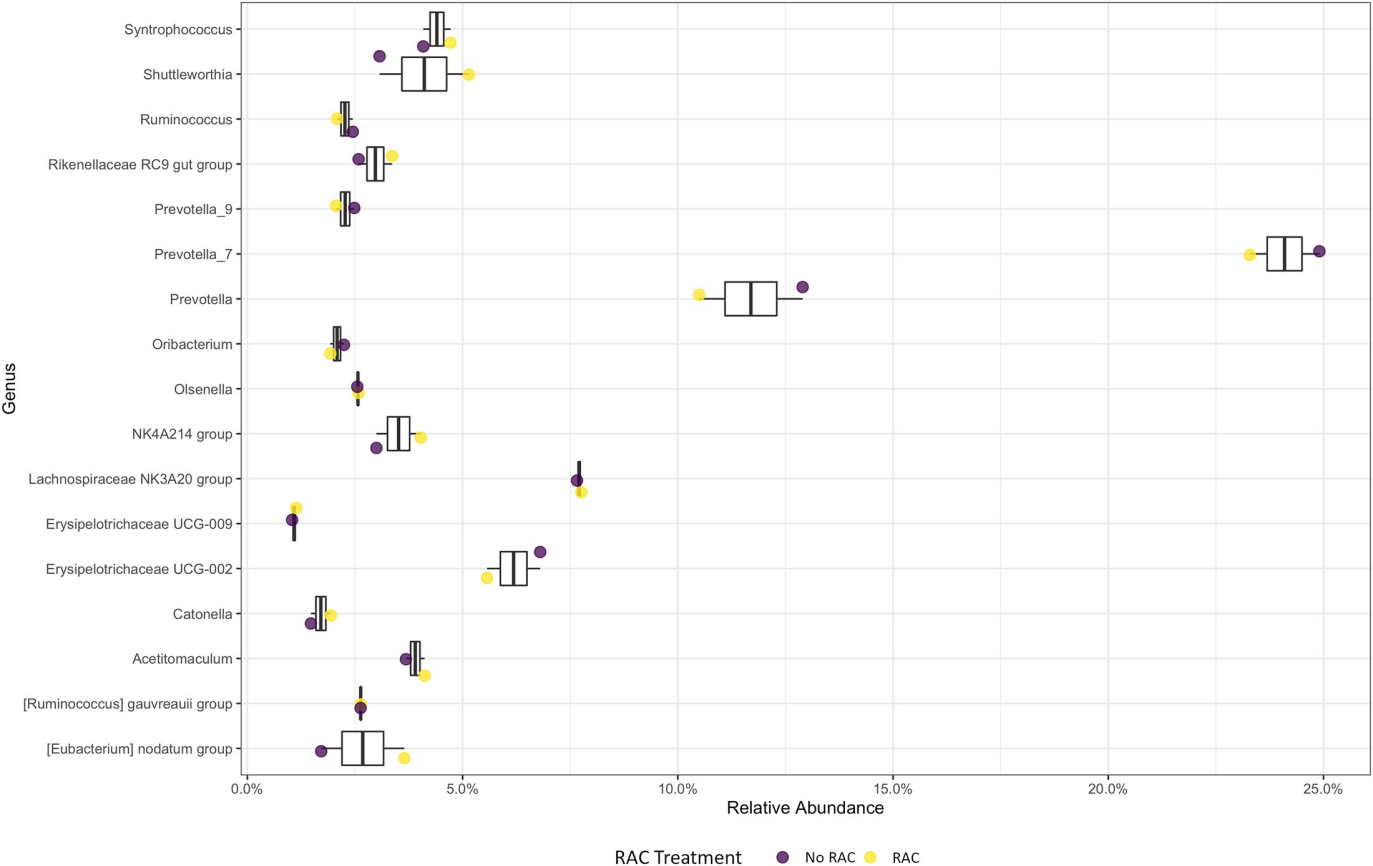
Taxonomic profile of the 99% genera relative abundance of the ruminal bacterial communities between the RAC concentration. The boxplot represents the genus-level abundances across all samples and different colored points represent different diets. Purple represents no ractopamine hydrochloride (RAC) added to the diet and yellow represents RAC added to the diet.

The 16S amplicon reads were analyzed to reflect the mean of the relative abundance (reads of the taxon/total reads in the sample) among the 6 treatments. The differential abundance based on dietary treatment (2×3 factorial design) analyzed using PROC MIXED in SAS v9.4 did not show significant differences at the phylum level. Genera abundances were not different across treatments for the interaction of protein and RAC (*P* > 0.05). However, there were differences when examining the main effects of protein and RAC (Figs 5 and 6). Genera *Ruminococcus gauvreauii group* (*P* = 0.005), *UCG_001* (*P* = 0.04), *UCG_002* (*P* = 0.015), *Ruminococcus* (*P* = 0.04) and *Eubacterium nodatum group* (*P* = 0.02) indicated significant differences in abundance between protein concentrations (Table 3). *Eubacterium nodatum group* showed significant differences in abundance with the RAC treatments (*P* = 0.016; Table 4).

**Table 3:** Percentage relative abundance of significant genera among protein concentration (CON, RDP, and RUP).

Genus	Protein Treatments^1^^,^^2^^,^^3^			SEM	*P* value^4^
	CON	RDP	RUP		
*Eubacterium nodatum group*	0.7341^a^	2.995^b^	2.225^ab^	0.005	0.021
*Ruminococcus gauvreauii group*	1.972^a^	1.26^b^	2.216^a^	0.0017	0.005
*Ruminococcus*	2.591^a^	1.374^ab^	1.049^b^	0.0046	0.041
*UCG -001*	0.5735^ab^	1.063^a^	0.1729^b^	0.0026	0.045
*UCG -002*	0.3961^b^	0.8574^a^	0.4194^b^	0.0012	0.015

^1^Values indicate the percent (%) relative abundance.

^2^Control diet (CON), rumen degradable protein diet (RDP), and rumen undegradable protein diet (RUP)

^3^Different letters indicate statistically significant differences.

^4^Significance difference determined at *P*<0.05.

**Table 4:** Percentage relative abundance of significant genera between RAC concentration (0 mg and 400 mg).

Relative genus abundance	RAC treatment^1^^,^^2^		SEM	*P* value^3^
	RAC	No RAC		
*Eubacterium nodatum group*	2.781	1.189	0.004	0.016

^1^Values indicate the percent (%) relative abundance.

^2^Ractopamine hydrochloride (RAC)

^3^Significance difference determined at *P*<0.05.

## 4 Discussion

The extensive use of beta-adrenergic agonists in the beef industry has resulted in positive improvements in the efficiency of body weight gain, carcass characteristics, and improving the competitiveness of the U.S. beef industry [9, 32]. As reported previously by Samuelson [2], the heifers of this study supplemented with 400 mg of RAC daily had greater final body weight (1.9% increase) and carcass-adjusted ADG (42.9% increase) contrasted to animals without supplementation [3]. The ADG results observed by Samuelson et al. [3] were greater than results reported by Abney et al. [33], where animals received just 200 mg of RAC daily [33]. Due to the increase of ADG in heifers with RAC treatment, metabolic protein requirements also increased to accommodate increasing gain. Both diet composition and differing feed intake levels impact metabolizable energy, generating a direct effect in the gain composition [34, 35].

Rumen degradable protein (RDP) and rumen undegradable protein (RUP) are different fractions of dietary protein supplied in the diet of ruminants. The site of digestion of those protein sources is different: RDP will be degraded by ruminal microorganisms, whereas RUP will escape the rumen to be digested and absorbed in the abomasum and small intestine [36]. In this study, several genera were significantly different between the protein concentrations (CON, RDP and RUP) in the diet (*Ruminococcus*, *Ruminococcus gauvreauii* group, *Eubacterium nodatum* group, *UCG-001* and *UCG-002*). These different organisms are well-known bacterial groups present in the rumen of cattle [37] that also are non-cellulolytic *Ruminococcus* species within the Ruminococcaceae family [38, 39]. Changes in rumen bacterial communities were anticipated, given that observations have shown increasing dietary protein and increases in highly fermentable substrates decreases the diversity of the rumen microbes, and increases the efficiency of those microbes to utilize these aforementioned substrates and drive rumen microbial structure [40].

Studies by Walker et al. [16] demonstrated that use of beta-adrenergic agonists in ruminants increased nitrogen retention and uptake of amino acids [16]. Specifically, they speculated that ractopamine increased post-absorptive amino acid uptake by peripheral tissues because total alpha-amino N decreased in the blood [16]. Although we hypothesized beta-adrenergic agonists in addition to different concentrations of dietary crude protein fractions would affect microbial communities in finishing beef heifers, we did not observe any meaningful differences. The bacterial communities of the rumen did not differ among the interaction of RAC and protein concentration. Specifically, there were limited impacts from RAC supplementation alone, which was noteworthy given that beta agonists share similarities with catecholamines, which have been shown to increase bacterial growth [12]. No significant differences were found when examining alpha-diversity (within-sample diversity). However, beta-diversity (between sample diversity) results indicated significant differences among the dietary crude protein (CON, RDP and RUP) and RAC. However, this difference was likely observed due to differences in the dispersions among samples. Statistical analyses and PCoA visualization do not always correspond if there is a weak separation of treatment groups. The PCoA visualization only captured approximately 18.6% of the variation. Thus, differences may be more evident on a different axis. The microbial communities of this study were similar in microbial composition to existing research, where Bacteroidetes and Firmicutes were the most abundant phyla among the treatments with RAC [41, 42]. Additionally, previous work has affirmed greater abundance of Bacteroidetes and Firmicutes over other phyla which is connected with the increase of ADG and could potentially affect feed efficiency [43].

Feeding behavior may impact the lack of RAC results noted in the current study. Of interest is the potential effect of heterogenous RAC intake within the collective pen, as RAC was administered on top of the protein treatments. Indeed, when supplemented with RAC at 300 mg/d for 28 d, steers had a greater number of daily feeding events and those events were in shorter duration [44]. In contrast, when feeding RAC over a longer 42 d period, visit eating time, per meal, and per day were reduced in steers [45]. Further, when RAC was supplemented at 200 mg/d for 30 d, steers had an extended time required to consume daily feed [33]. However, although the rates of dry matter intake (DMI) may have been impacted in several studies, overall DMI in this study [3] and others [45] was not different based on RAC treatment. As rumen microbiome function and structure are well documented to be impacted by diet composition and DMI [43, 46], it is unlikely modifications in feeding behavior based on RAC treatment contributed to the observed results in this study.

Different studies confirm the effect of adrenergic agonists on the contractions in the rumen [47–49]. Adrenergic agonists reduce the frequency and intensity of the ruminal contractions, producing an effect on the digestion of the nutrients from the diet by the ruminal microbial community [48, 49]. Normally, this effect is caused by alpha-adrenergic agonists (α-AA), such as phentolamine, which presents an affinity with alpha-adrenergic receptors (α-AR). By the activation of these α-AR by the α-AA, the receptors produce an inhibitory effect, releasing acetylcholine from the parasympathetic postganglionic nervous terminals. The sympathetic system response of these reactions is an indirect inhibition of the gastrointestinal functions and motility [50]. Additionally, these interactions reduce the frequency and amplitude of reticulum and rumen dorsal sac contraction, decreasing the diameter of the arteriole and increasing the resistance to blood flow. These receptors are present on the arterioles of the skin and the splanchnic vasculature [50]. In this study, we supplied in the diet a beta-adrenergic agonist, RAC, which as mentioned above, has an affinity for beta adrenergic receptors, suggesting that the reason we may not have observed any effect of RAC in the rumen may be explained by the differing affinity to alpha- or beta-adrenergic receptors.

Due to the demonstrated effects of natural catecholamines on bacterial growth [12], this study hypothesized that beta-adrenergic agonists, as synthetic catecholamines, could impact the rumen microbial communities. However, there was no impact of RAC on the rumen microbial communities. Although the supplementation with RAC or different protein sources, such as RDP and RUP, altered specific microbes of the rumen bacteria composition, the overall microbial population was not affected by the experimental treatments. The lack of effect of beta-adrenergic agonists on the rumen microbes may be due to absorption. The oral administration of RAC results in absorption from the gastrointestinal tract by the β2-AR, with a significant portion reaching systemic circulation. In cattle and monkeys, this amount reaching circulation is approximately >45%, whereas in swine, it has been documented to be >85% [51]. These circulation data suggest that beta-adrenergic agonists may have the same characteristics as the RUP, which escape the rumen and are digested and absorbed in the small intestine. Therefore, the administration of beta-adrenergic agonists may not interact or affect the microorganisms in the rumen and escape for absorption in the small intestine. Subsequently in the hindgut, RAC may play the same role as catecholamines regarding bacteria and the necessity of those to obtain iron for its growth.

Further, beta-adrenergic agonists are lipophilic, presenting aliphatic amine compounds with an alkaline pKa ranging from 9.4 for RAC. However, at physiologic pH, RAC is ionized by protonation and more than 99% will be positively charged at a pH closer to 7.4, creating a non-lipophilic compound that would be activated just with the specific receptor [51]. The absorption location of the beta-adrenergic agonists is not clear. Different types of beta-adrenergic agonists have shown absorption in the small intestine and less absorption in the stomach in experiments with rats and absorption in the duodenum in humans. However, due to the limited information regarding the absorption site of beta-adrenergic agonists in ruminants in vivo, it is possible to hypothesize that the site of the primary absorption of beta-adrenergic agonists in ruminants is in the small intestine. This absorption could be through passive diffusion, as a result of the neutral pH in the small intestine preventing beta-adrenergic agonists to form cations at the phenethanolamine nitrogen, increasing the absorption through the intestinal mucosa [52].

The results in this study suggest that the action of RAC and different concentrations of protein may not be synergistic when acting upon the rumen bacterial communities, suggesting the supplements function independently. Therefore, with the known activity of beta-adrenergic receptors in the regulation of gastrointestinal functions, such as the secretion of bicarbonate and acid, intestinal motility, and gastrointestinal mucosal blood flow, future studies should examine the relationship of beta-adrenergic agonists with beta-adrenergic receptors present in cattle gastrointestinal tracts and in the rumen to obtain a better understanding of the mediation of beta-adrenergic receptors in bovine physiological responses [53–55].
